# Editorial: Induced cell senescence as a therapeutic strategy for cancer treatment

**DOI:** 10.3389/fonc.2022.1104877

**Published:** 2022-12-07

**Authors:** Kaiyue He, Liyuan Lu, Yong-Ping Jian, Zhi-Xiang Xu

**Affiliations:** ^1^ School of Life Sciences, Henan University, Kaifeng, Henan, China; ^2^ Department of Medicine, University of Alabama at Birmingham, Birmingham, AL, United States

**Keywords:** cell senescence, cell-cycle arrest, tumor suppressor genes, cancer treatment, tumor nanotherapeutics

Cell senescence is an irreversible natural state of cell-cycle arrest, which is induced by various signals of intracellular or extracellular stress stimuli (1, 2). The characteristics of cell senescence include cell-cycle withdrawal, macromolecular damage, secretory phenotype and cell metabolism disorder, which are interdependent (1). As a cellular defense mechanism, senescence occurs in different physiological and pathological processes, including tissue remodeling, injury, cancer and aging (3). Cell senescence is generally considered to be a tumor-suppressive mechanism, overcoming of which plays a critical role in cancer development and progression (2). Tumor suppressor genes, such as p53, p21, p27 and Rb, regulate cell senescence. However, the mechanisms by which tumor cells overcome cell senescence and maintain the transformative state are still elusive. In addition, how to induce tumor cell senescence to serve as a therapeutic strategy for cancer treatment is under extensively explored. Thus, studies of the molecular mechanisms associated to cell senescence and the escape of cancer cells from senescence will provide a new perspective for the therapeutic application of cancer.

P53-p21 (CIP1/WAF1/SDI1) signal is considered as a classical pathway to induce cell senescence (4). In this Research Topic, Chen et al. utilized lung cancer cell line and transgenic mouse model of lung cancer to study the effects of GATA6 on the growth of lung cancer cells and its potential working molecular mechanism. The authors demonstrated that GATA6 induces cell senescence in lung cancer by up-regulating p21 and p53 and inhibiting AKT, thus suppressing the growth of lung cancer. Ectopic expression of GATA6 significantly inhibited xenografted lung tumor growth (**Figure 1**). Therefore, the recovery of GATA6 expression may provide an effective treatment strategy for lung cancer patients with GATA6 deficiency.

Wnt/β-catenin signaling pathway is also associated with tumor cell senescence (5, 6). In this Research Topic series, two groups reported that tumor suppressor ZNF24 induces the senescence of non-small cell lung cancer (NSCLC) and thyroid cancer (THCA) cells (Pang et al., Xiong et al.). ZNF24 inhibits WNT signaling pathway by preventing β-catenin to form a complex with TCF1/LEF1 and ultimately induces tumor cell senescence (**Figure 1**). Ectopic expression of ZNF24 markedly inhibited the growth of THCA and NSCLC tumors. Transgenic mouse models also confirmed that expression of ZNF24 inhibited the occurrence and development of NSCLC. ZNF24 is highly deficient in NSCLC and THCA. Thus Wnt inhibitors, such as ICG-001, MSAB, and 2,4-Dia, are likely to provide treatment opportunities for NSCLC and THCA patients with ZNF24 deficiency.

Metabolic reprogramming is one of the phenotypic changes experienced by senescent cells (7). Zhang et al. found that TFCP2 inhibited the synthesis of cholesterol and hence suppressed the senescence of pancreatic cancer cell. The authors demonstrated that TFCP2 interacts with sterol regulatory element binding transcription factor 2 (SREBP2) to synergistically activate the expression of HMGCR, a rate limiting enzyme for cholesterol synthesis, and inhibit the senescence of tumor cells (**Figure 1**). Application of HMGCR inhibitor statins effectively reverses the inhibitory effect of TFCP2 on senescence. The study highlights a new mechanism for TFCP2-suppressed senescence in pancreatic cancer cells, which may provide a new strategy for the treatment of pancreatic cancer by suppression of TFCP2 signaling and induction of senescence.

Senescent cells send signals to and affect their surroundings although senescence is an internal cellular process (7). Senescent cancer cells produce a senescence-associated secretory phenotype (SASP), which recruits and activates immune cells to induce anti-tumor immunity (8), whereas senescent cancer cells bear a strong immunogenicity. Using senescent cancer cells as cancer vaccines can enhance the ability to send signals to or receive signals from tumor microenvironment (TME), induce the processing and presentation of MHC-I antigens, and ultimately activate the adaptive anti-tumor immunity mediated by DCs and CD8+ T cells (**Figure 1**) (9). Hu et al. conducted a comprehensive analysis of cell senescence related genes in LUSC by using The Cancer Genome Atlas (TCGA) and Gene Expression Omnibus (GEO), and established a new risk model to predict the prognosis of LUSC and response to immunotherapy. It showed that senescence related-genes control immune cell infiltration of TME and responses to immune checkpoint blockage (ICB) therapy. Therefore, it is a promising strategy to interfere with key factors regulating immune response, which can improve the clearance of precancerous senescence cells in high-risk LUSC and inhibit tumor growth.

**Figure 1:**
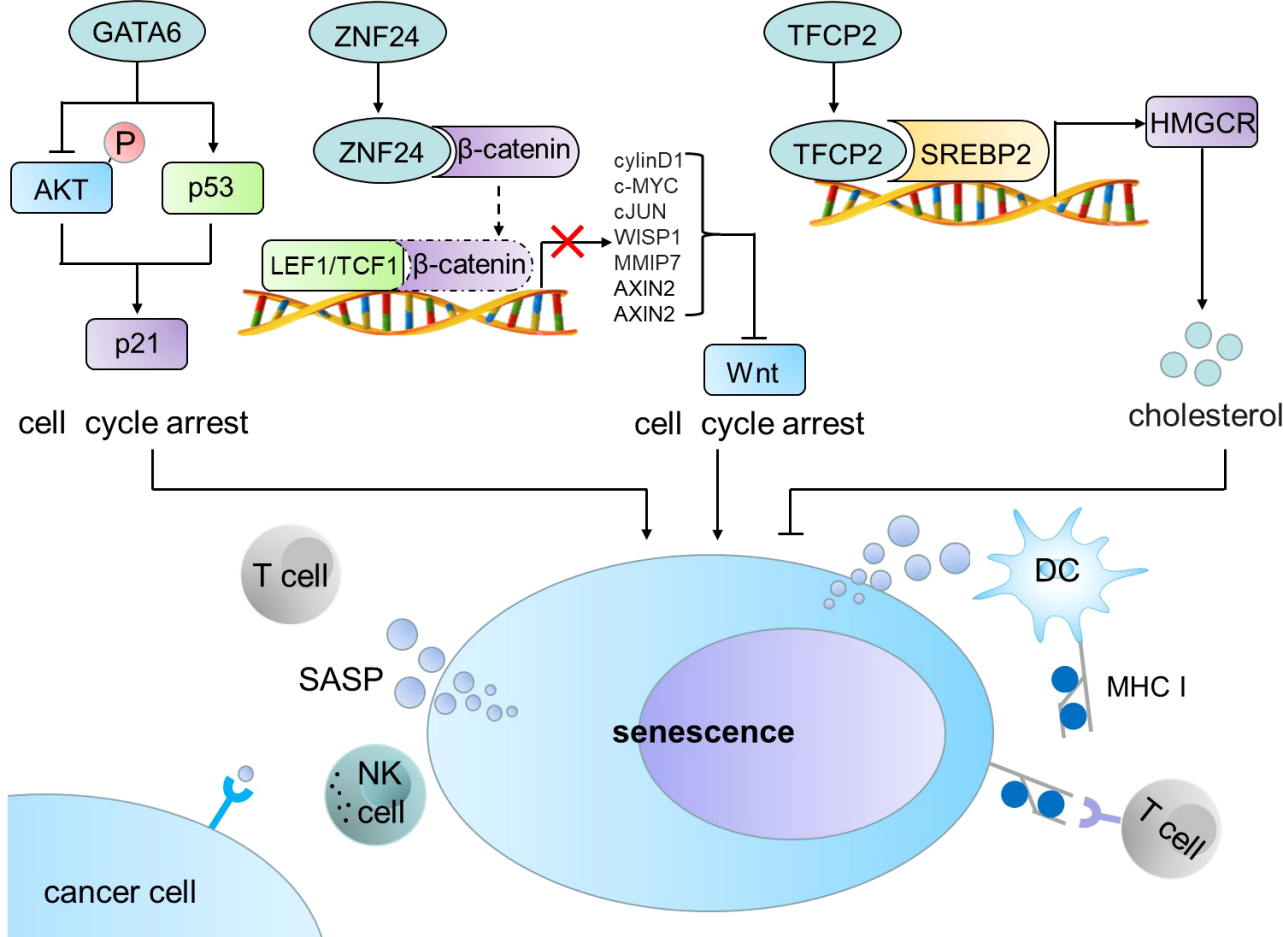
GATA6, ZNF24 and TFCP2 induce cell senescence and trigger anti-tumor immune response. GATA6 induces cell senescence in cancer cells by up-regulating p21 and p53 and inhibiting AKT. ZNF24 inhibits WNT signaling pathway by preventing β-catenin to form a complex with TCF1/LEF1 and ultimately induces tumor cell senescence. TFCP2 interacts with SREBP2 to synergistically activate the expression of HMGCR and inhibit the senescence of tumor cells. Senescent tumor cells produce SASP, which can recruit and activate immune cells in TME to induce anti-tumor immunity. SREBP2, sterol regulatory element binding transcription factor 2; SASP, senescence-associated secretory phenotype; DC, dendritic cell; MHC I, major histocompatibility complex I.

Although treatment-induced senescence is an anti-tumor mechanism initially, long-term treatment-induced senescence of cells may be harmful. Long-term induction of senescence will produce a microenvironment that promotes inflammation and immunosuppression (10). Therefore, induction of cell senescence as a therapeutic strategy for cancer treatment should be context-dependent and evaluated carefully based on the cancer stage. It is also an option to develop new therapeutic strategy to combine application of induced-senescence with other treatments.

Over the past decade, immunotherapy has witnessed a new time for an effective and promising cancer therapy (11). Immunotherapy shows the potential to improve the efficacy of cancer therapy, reduce side effects and inhibit tumor recurrence (12). However, the clinical responses of cancer patients to immunotherapy, in particular to ICB treatment, are relatively low (13, 14). This is mainly due to low immunogenicity, tumor heterogeneity, and immunosuppressive TME, which lead to the amplification of tumor burden (15, 16). Induced senescence could enhance immune cell infiltration to TME and promote responses to ICB by enhancing immunogenicity (Hu et al).

Tumor nanotherapeutics provides a new strategy for fighting cancer related drug resistance and tumor recurrence, especially for overcoming the limitations of traditional tumor immunotherapy (17, 18). In the review article of this serial, Katopodi et al. discussed the research progress of nanomedicine engineering for combinational tumor-targeted immunotherapy. In order to overcome the obstacles of high toxicity and drug resistance of traditional chemotherapy/immunotherapy, nanotherapeutics can precisely target tumors,cancer stem-like cells (CSCs) and TME. On one hand, based on the unique magnetic and optical properties, nanoparticles are able to be used as photothermal agents to kill cancer cells. On the other hand, exosomes, nanogels and microneedles are used as nanocarriers, which are combined with micro-RNA, tumor antigen, immunotherapy and self-assembly prodrug as nanotherapeutic strategies. The application of nanotherapeutics can not only reduce the drug toxicity, keep the drug concentration in tissues, blood and specific target areas stable, but also enhance the drug targeting and overcome tumor immune resistance. Currently, clinical application of nanotherapeutics is relatively small although it bears multiple advantages over the routine cancer therapy. Hurdles for the wide application of nanotherapeutics in cancer treatment are the production of large-scale, replicable commercial batches of nanomedical formulations with improved efficacy and decreased toxicity.

In summary, senescence is induced in cancer cells through different mechanisms. Induced senescence could activate immune cells to act as an anti-tumor mechanism. ICB and nanotherapeutics can overcome the disadvantages of traditional cancer therapy, such as severe side effects, lacking of responses, drug resistance and high recurrence rate. Thus, inducing cell senescence and emerging cancer ICB and nanotherapeutics have opened up new avenues for cancer treatment.

## Author contributions

KH and LL wrote the manuscript. Y-PJ and Z-XX contributed to the conception and writing. All authors read and approved the final manuscript.
